# Patient's Awareness of Cancer-Associated Thrombosis: A Canadian Nationwide Survey

**DOI:** 10.1055/a-2635-9296

**Published:** 2025-07-07

**Authors:** Ana C. Pizzarossa, Andrea Penaloza, Kristina Vrotniakaite-Bajerciene, Rufaro Chitsike, Vicky Tagalakis, Susan Calverley, Marc Carrier

**Affiliations:** 1 Hemostasis and Thrombosis Unit, Hospital de Clínicas “Dr Manuel Quintela,” Montevideo, Uruguay; 2 Department of Medicine, University of Ottawa at the Ottawa Hospital and The Ottawa Hospital Research Institute, Ontario, Canada; 3 Emergency Department, Cliniques Universitaires Saint-Luc, Université Catholique de Louvain, Brussels, Belgium; 4 Department of Hematology and Central Hematology Laboratory, Inselspital, Bern University Hospital, University of Bern, Bern, Switzerland; 5 Division of Medicine (Hematology), Memorial University of Newfoundland, St John's, Newfoundland and Labrador, Canada; 6 Department of Medicine, Jewish General Hospital, Montréal, Québec, Canada; 7 Thrombosis Canada, Ontario, Canada

**Keywords:** venous thrombosis, venous thromboembolism, pulmonary embolism, neoplasias, anticoagulants

## Abstract

**Background:**

Approximately 20% of patients with cancer will have cancer-associated venous thromboembolism (CAT), which is associated with significant morbidity and mortality. Despite its clinical importance, CAT awareness in cancer patients and caregivers remains low. We sought to assess the patients' knowledge of CAT through a national survey.

**Materials and Methods:**

A survey assessing knowledge of different aspects of CAT was developed by a steering committee including four clinicians with expertise in CAT and a patient partner with lived experience. Survey dissemination among patients with cancer occurred through the Environics network, the Thrombosis Canada member network, the Thrombosis Canada social media platforms, and was advertised through Instagram and Facebook, and the Canadian Cancer Survivor Network newsletter.

**Results:**

Out of the 312 patients with cancer or survivors who responded to the survey, 179 (57.4%) were female, and 118 (37.8%) were over 65 years old. Overall, 119 patients (38.1%, 95% confidence interval [CI]: 37.7–49.8%) reported having no knowledge of CAT. Only 84 (26.9%, 95% CI: 22.1–32.2%) and 94 (30.1%, 95% CI: 25.1–35.6%) patients reported receiving education about their underlying risk of CAT or education about signs and symptoms of venous thromboembolism, respectively. A total of 66 (21%, 95% CI: 16.8–26.1%) patients reported being informed by a health care professional about considering thromboprophylaxis. Patients were interested in learning more about the risk of CAT, its associated risk factors, and the benefits and potential side effects of thromboprophylaxis.

**Conclusion:**

Many patients with cancer lack awareness or knowledge of CAT. Our results highlight ongoing education and awareness of the CAT burden.

## Introduction

Venous thromboembolism (VTE) consists of the interrelated diseases of pulmonary embolism (PE) and deep vein thrombosis (DVT). VTE is estimated to affect 1 to 2 individuals per 1,000 person-years in Europe and the United States, with lower rates in other regions. 1 Cancer and its various treatments are well-recognized risk factors for VTE. It is estimated that patients with cancer have a four- to fivefold higher risk of VTE compared to the general population. Hence, approximately 20% of these patients will have cancer-associated venous thromboembolism (CAT). 2 3 Of these, over 50% will occur in the first 3 months following the cancer diagnosis, complicating its management (e.g., delaying procedures or surgeries, etc.). Furthermore, CAT remains a major cause of morbidity and mortality in this population. 4 5 Despite its frequent occurrence and clinical importance, CAT awareness in cancer patients and caregivers seems to remain low. 6 7 Patients with cancer have little knowledge of CAT, and most receive no information about CAT, despite its distressing impact on them and their families. 8 Previous surveys show that patients are interested in receiving education on signs and symptoms of VTE, risk factors for CAT, and information on prevention strategies. 9 10 Hence, there is a persisting knowledge gap among patients with cancer regarding CAT. We aim to better characterize this gap by assessing Canadian patients' awareness and knowledge about different components of CAT (e.g., risk factors, signs and symptoms, thromboprophylaxis, etc.).

## Methods

We conducted a cross-sectional study using a self-administered questionnaire completed by participants ( Supplementary Material ). The survey was conducted by Environics Research, an independent research company, and targeted Canadian patients with cancer and cancer survivors (see survey in Supplementary Material ). The electronic survey was designed using established methods. 11 The survey was developed by a steering committee including four clinicians specialized in Adult Thrombosis Medicine and one patient partner with lived experience. The research was exempt from research ethics review as it involved anonymous participation in an online survey. Since the survey is anonymous, the confidentiality of the respondents' answers was preserved.

Our initial step involved screening questions to ascertain participant eligibility (patient and cancer demographics). The main body of the survey assessed awareness and knowledge of common aspects of CAT (e.g., risk factors, signs and symptoms, anticoagulants for thromboprophylaxis and treatment, etc.). These questions were strategically selected to assess all components of CAT and determine the baseline knowledge, which will enable conversations about CAT between patients and health care professionals. Additionally, we included questions to gauge interest in education related to CAT, along with preferred topics and format. The survey was available in both English and French. To ensure the survey's validity and clarity, we piloted it in both physicians and patients, leading to subsequent revisions.

We adopted a multifaceted approach for survey dissemination, utilizing email and social media. Survey dissemination occurred through the Environics network, the Thrombosis Canada member network, Thrombosis Canada social media posting, and advertising through Instagram and Facebook. Additionally, we reached out to the patient group, the Canadian Cancer Survivor Network, for distribution through their newsletter. These societies were strategically chosen for their representation of physicians who provide medical care to patients with CAT, patients with cancer, and cancer survivors.

Data for this study were collected between May and June 2024 using the SurveyMonkey platform. To optimize response rates, we employed several strategies, including the design of a straightforward questionnaire using lay terminology. Additionally, participants were contacted multiple times to encourage survey completion. 12 Descriptive statistics were utilized to summarize respondent counts and proportions for each question.

## Results

Of the 312 patients with cancer or survivors surveyed, 179 (57.4%) were female and 118 (37.8%) were over 65 years old. Responses were received from all 10 provinces of Canada, with most from Ontario (116, 37.3%) and Quebec (53, 16.9%). Baseline characteristics of respondents are depicted in Table 1 . A total of 219 (70.2%) had active cancer, whereas 93 (29.8%) were cancer survivors, and 181 (58.1%) reported having post-secondary education. The most frequent primary cancer sites were breast (84, 26.9%) and prostate (46, 14.7%; Table 1 ). Overall, 239 (76.6%) patients were receiving or had received cancer treatments. A total of 85 (27.2%) patients reported a prior history of VTE. Of these, 21 (6.7%) occurred before the cancer diagnosis, 24 (7.7%) after diagnosis but before treatment, and 40 (12.8%) during cancer treatment. Of the 85 patients with VTE, 46 (54.1%) reported that thrombosis had a negative impact on their quality of life, and 70 patients (82.4%, 95% confidence interval [CI]: 72.6–89.8%) would have wanted information about CAT before it occurred.

**Table 1:** Patients' demographic and cancer characteristics

Characteristic	Percentage ( *N* )
Sex	
Female	57.4 (179)
Male	42.6 (133)
Age (years)	
18–39	10.9 (34)
40–64	51.3 (160)
≥65	37.8 (118)
Region of residence	
Ontario	37.3 (116)
Quebec	16.9 (53)
British Columbia	14.0 (44)
Alberta	12.0 (37)
Manitoba/Saskatchewan	11.4 (36)
Atlantic	8.4 (26)
Education	
Not finished high school	6.2 (19)
Finished high school	28.1 (88)
Post-secondary	58.1 (181)
Other	7.1 (22)
First language	
English	73.9 (231)
French	19.1 (68)
Other	7.0 (25)
Primary cancer location	
Breast	26.9 (84)
Prostate	14.7 (46)
Skin without melanoma	10.3 (32)
Lung	9.6 (30)
Colorectal	9.3 (29)
Gynecologic (uterus, cervix, vagina)	8.7 (27)
Bladder	6.4 (20)
Hematologic (excluding lymphoma)	5.8 (18)
Kidney	5.8 (18)
Lymphoma	5.4 (17)
Melanoma	3.8 (12)
Liver	3.8 (12)
Brain	2.9 (9)
Thyroid	2.6 (9)
Pancreas	2.6 (8)
Stomach or esophagus	2.6 (8)
Sarcoma	2.2 (7)
Other	2.2 (7)
Status of cancer treatment	
Treatment starting soon	5.4 (17)
Ongoing	38.1 (119)
Completed	38.5 (120)
Watch and wait	16.3 (51)
Not sure	1.6 (5)
Type of cancer treatment (ongoing or completed)	
Surgery	46.5 (145)
Chemotherapy	41.7 (130)
Radiation therapy	39.7 (124)
Hormone therapy	15.7 (49)
Immunotherapy	14.7 (46)
Targeted therapy	13.8 (43)
Transplant	6.1 (19)
Drug therapy	1.6 (5)
Other	3.5 (11)
None of the above	4.8 (15)
Not sure of the type	2.2 (7)

**Fig. 1:**
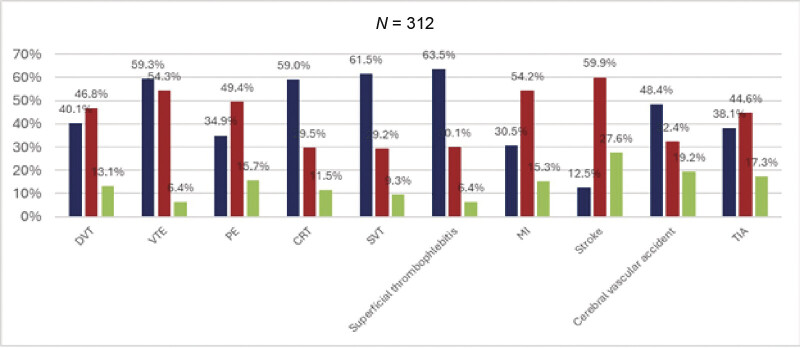
Familiarity with terms related to venous and arterial thrombosis. Respondents had to choose a number between 1 to 7 according to their familiarity with the terms proposed. Scale was 1 = no knowledge to 7 = very knowledgeable. Summary chart legend is green (very knowledgeable) = top two (6 and 7); red (somewhat) = middle three (3–5); blue (very little/no knowledge) = bottom two (1 and 2). CRT, catheter-related thrombosis; DVT, deep vein thrombosis; MI, myocardial infarction; PE, pulmonary embolism; SVT, superficial vein thrombosis; TIA, transient ischemic attack; VTE, venous thromboembolism.

**Fig. 2:**
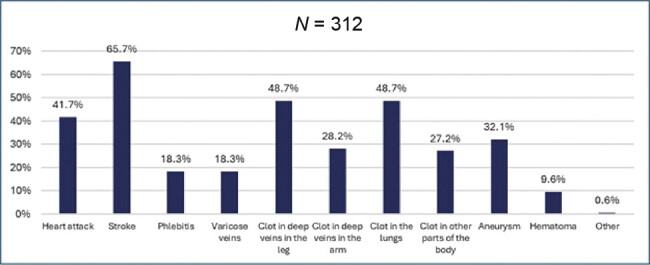
When you think of blood clots, which of the following do you think of? Respondents had to choose which terms they associated with blood clots.

**Fig. 3:**
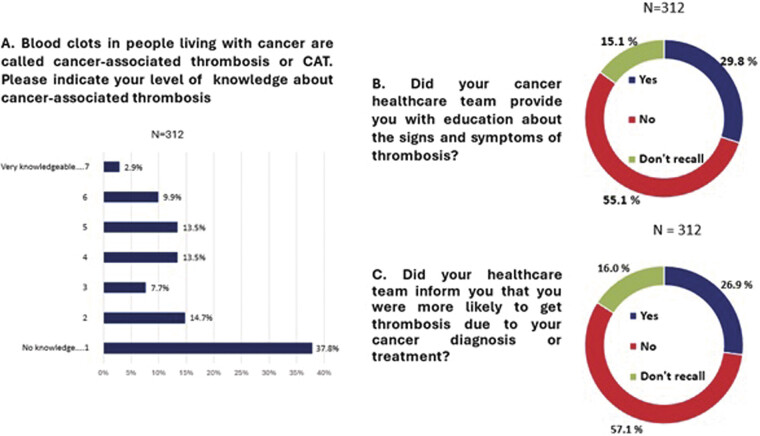
Awareness of cancer associated thrombosis. Respondents had to choose a number between 1 to 7 according to their familiarity with the terms proposed. Scale was 1 = no knowledge to 7 = very knowledgeable. Summary chart legend is: Blue (Very knowledgeable) = Top 2 (6 and 7); Red (Somewhat) = Middle 3 (3,4,5); Green (Very Little/no knowledge) = Bottom 2 (1,2).

Knowledge about CAT was low among patients with cancer and cancer survivors. Overall, 125 (40.1%, 95% CI: 34.6–45.5%) and 109 patients (34.9%, 95% CI: 29.7–40.2%) reported having very little or no knowledge of DVT or PE, respectively. Interestingly, only 39 (12.5%, 95% CI: 8.8–16.2%) respondents had very little or no knowledge on stroke ( Fig. 1 ). Respondents associated blood clots with DVT in 49% (95% CI: 43.4–54.7%) and stroke in 66% (95% CI: 6,205–7,301%) of cases ( Fig. 2 ). Overall, a majority of respondents (164, 52.5%, 95% CI: 46.9–58.2%) reported that they had very little or no knowledge about CAT ( Fig. 3 ). A total of 119 patients (38.1%, 95% CI: 37.7–49.8%) reported having no knowledge of CAT. Only 84 (26.9%, 95% CI: 22.1–32.2%) and 93 (29.8%, 95% CI: 24.8–35.2%) patients reported receiving education on CAT risk or VTE symptoms or signs by their health care team, respectively. Among patients informed about CAT risk, 43 (51.2%, 95% CI: 40.0–62.3%) were informed during cancer treatment, and only 7 (8.3%, 95% CI: 3.4–16.4%) received information before the initiation of chemotherapy or cancer treatment. Of the patients reporting having received education about the signs and symptoms of CAT, 79 (84.0%, 95% CI: 75.1–90.8%) received instructions on what to do or whom to contact if they recognized those symptoms. Patients with a prior VTE had greater knowledge of CAT (e.g., awareness of the term CAT, etc.). No difference in knowledge of CAT was observed between participants who did not complete high school and those with higher levels of education.

Awareness about risk factors or signs and symptoms of CAT was low among respondents. A total of 114 (36.5%, 95% CI: 31.2–42.2%) of respondents were unaware of any risk factors for CAT. The most commonly recognized risk factors were reduced physical activity (109, 34.9%, 95% CI: 29.7–40.5%) and recent surgery (79, 25.3%, 95% CI: 20.6–30.5; Supplementary Fig. S1 ). Among respondents, 158 (50.6%, 95% CI: 45.0–56.3%) patients with cancer and cancer survivors recognized leg swelling as a sign of DVT and 78 (25.0%, 95% CI: 20.3–30.2%) and 66 (21.2%, 95% CI: 16.8–26.1%) identified shortness of breath and chest pain, respectively, as symptoms of PE. Overall, 82 (26.3%, 95% CI: 21.5–31.5%) respondents were uncertain whether any of the listed symptoms and signs were related to CAT ( Supplementary Fig. S2 ).

Most patients with cancer or cancer survivors felt somewhat or very knowledgeable about blood thinners (269, 86.3%, 95% CI: 81.9–89.8%) and anticoagulants (202, 64.7%, 95% CI: 59.2–70.0%). Similarly, 185 (59.3%, 95% CI: 53.6–64.8%) and 166 (53.2%, 95% CI: 47.5–58.9%) were familiar and knowledgeable about warfarin and heparin, respectively ( Supplementary Fig. S3 ). Approximately 66 respondents (21%, 95% CI: 16.8–26.1%) reported being advised by a health care professional to consider thromboprophylaxis. Among the respondents, 157 (50.3%) reported prior or current use of an anticoagulant, with 128 (41.0%) and 78 (25.0%) patients having used or using an oral or a parenteral agent, respectively. Interestingly, of those prescribed an anticoagulant, only 67 (42.8%, 95% CI: 34.8–50.8%) recalled being informed by a health care provider of its necessity. Furthermore, only 62 (39.4%, 95% CI: 31.8–47.6%) and 55 (35.0%, 95% CI: 27.6–43.0%) respondents were informed of possible side effects and potential benefits of taking anticoagulants, respectively. Finally, 82 (52.2%) patients who have taken or are taking anticoagulants reported worrying about bleeding complications, and 54 (34.3%) reported worrying about new thrombosis.

A large majority of respondents rated education on CAT as important information to have following the diagnosis of cancer ( Supplementary Fig. S4 ). A total of 210 (67.3%, 95% CI: 61.8–72.5%) patients with cancer or survivors felt that it is very important for the health care team to provide information and education about CAT. The topics of most interest were ( Supplementary Table S1 ): (1) Risk of CAT with cancer (133, 42.6%, 95% CI: 37.1–48.3%), (2) risk factors for CAT (120, 38.5%, 95% CI: 33.0–44.1%), and (3) side effects of anticoagulants (120, 38.5%, 95% CI: 33.0–44.1%). While websites are the most preferred format (175, 56.0%, 95% CI: 50.4–61.7%), there is also interest in paper brochures (119, 38.1%, 95% CI: 32.7–43.8%), videos (94, 30.1%, 95% CI: 32.7–43.8%), and downloadable brochures (81, 26.0%, 95% CI: 21.2–31.2%).

## Discussion

Our survey highlights a significant lack of awareness and education about CAT among Canadian patients with cancer and survivors of cancer. Over half of the respondents reported limited or no knowledge of CAT, and only one-third recalled receiving education from health care providers. Despite many patients having experienced CAT, 82.4% were not informed about it prior to their diagnosis. Importantly, 67% expressed a strong interest in receiving education about CAT, especially its risk factors, signs and symptoms, and the potential benefits and side effects of anticoagulation.

Our findings of low awareness of CAT are consistent with previous European and international studies 6 7 which report that only 27 to 38% of patients receive education on CAT. 6 7 This emphasizes the need for increased awareness about CAT worldwide. Similarly, these studies also identified better awareness of stroke than DVT or PE, suggesting a global knowledge gap for CAT. Compared to the European survey, our respondents were less likely to recognize symptoms of PE and more likely to attribute unrelated symptoms to VTE. This emphasizes the need for clearer, more accessible education to ensure that patients seek medical attention promptly and avoid potential additional morbidity related to a delayed diagnosis of CAT. A mixed-method study has previously demonstrated that a simple information video on CAT for patients receiving systemic cancer treatment was associated with reduced delays in diagnosis by 6 days, thereby reducing long-term complications. 13 Furthermore, the lack of awareness and knowledge about VTE seems to be a long-standing and persisting issue. An international survey conducted 10 years ago in the general population reported an important lack of knowledge about DVT (56%) and PE (46%), but not of stroke (15%). 14 Hence, there is an urgent need to create new knowledge mobilization strategies to address this important gap.

In our survey, patients with cancer considered receiving education on CAT to be very important, and only 21% of them reported being informed by a health care professional about thromboprophylaxis. Therefore, efforts to improve CAT education and prevention must go beyond awareness and focus on knowledge implementation. The Venous Thromboembolism Prevention in the Ambulatory Cancer Clinic (VTEPACC) 15 program is an effective multidisciplinary model demonstrating that structured risk assessment and tailored thrombosis education can improve patient outcomes and decision-making. The VTEPACC program reported an increase in VTE education and risk assessment rates from 5 to 95% in patients with cancer. A total of 23% of patients with cancer undergoing chemotherapy included in the study were identified to be at high risk of CAT using a risk assessment score (i.e., the Khorana score). These patients received education on risk factors, signs and symptoms of VTE, and consideration of pharmacological thromboprophylaxis. Overall, 94% of high-risk patients who received additional education and evaluation from the healthcare team elected to receive thromboprophylaxis with an anticoagulant. 15 Similarly, another study reported a practical, multidisciplinary model for implementing CAT prevention in a Portuguese outpatient setting. 16 Using the Khorana score to identify high-risk patients, the model includes systematic VTE risk assessment done by nursing, followed by specialist evaluation and appropriate thromboprophylaxis with direct oral anticoagulants or low-molecular-weight heparin. Among the 190 patients evaluated, thromboprophylaxis was administered to 86.5% of high-risk patients, with subsequent low rates of VTE (4.7%) and major bleeding (2.3%). These models have demonstrated that knowledge implementation is feasible and adaptable to clinical workflows. Both models are potentially eligible for broader adaptation to improve CAT awareness, knowledge, and prevention in patients with cancer. Furthermore, educational interventions have also been shown to improve treatment adherence in patients with a diagnosis of CAT. The implementation of a structured and personalized patient education program for CAT was shown to be feasible and improved cancer patient empowerment, adherence to CAT anticoagulant treatment, and quality of life. 17 18 Based on the survey findings, several initiatives are being developed to enhance awareness and education on CAT. Thrombosis Canada is planning to implement strategies such as the development of patient education materials, interactive tools, and individualized risk assessment resources (including a web-based Khorana Risk Score calculator). Additional initiatives include the creation of a podcast, an accredited webinar, and targeted awareness campaigns aimed at both patients and healthcare professionals, including an e-learning program for clinicians. Promoting active patient engagement in educational efforts is essential.

Our survey has several strengths, including its national scope, the inclusion of a patient partner in its development, and the participation of respondents with diverse cancer types and experiences. However, limitations include possible recall and selection biases, and underrepresentation of non-English/French speakers or those without internet access. Furthermore, our findings may not be generalizable to patients from other countries. Finally, as information on the survey was widely disseminated online, it was not possible to calculate a response rate.

## Conclusion

In conclusion, our survey reinforces that awareness and education about CAT among Canadian patients with cancer remain low. Patients are interested in learning about CAT and how to reduce their underlying risk. To address this gap, healthcare providers must prioritize timely and accessible education using multidisciplinary patient-centered strategies to ensure that CAT education is integrated along the cancer care pathway.
