# Supplementary material for: Patient's Awareness of Cancer-Associated Thrombosis: A Canadian Nationwide Survey
Source: TH Open. 2025 Jul 7;9:a26359296. doi: 10.1055/a-2635-9296 (PMC12265400; doi:10.1055/a-2635-9296)
Supplement: Supplementary file 1 — Supplementary Material [file 10-1055-a-2635-9296_26505331.pdf]

Supplementary Material

Screening and Initial Demographics

A. What is your province or territory of residence?
Select one response.
☐ Newfoundland and Labrador
☐ Prince Edward Island
☐ Nova Scotia
☐ New Brunswick
☐ Quebec
☐ Ontario
☐ Manitoba
☐ Saskatchewan
☐ Alberta
☐ British Columbia
☐ Yukon
☐ Northwest Territories
☐ Nunavut
☐ Other

MUST LIVE IN CANADA TO CONTINUE

B. To ensure we include people from all parts of Canada, what are the first three characters of your postal code?
Postal code: ___ ___ ___

C. In what year were you born?
Numeric dropdown ($<$1920 to $>$2003).
Prefer not to answer.

MUST BE $\geq$18 YEARS TO CONTINUE

D. Select the option that best describes your current gender identity.
Select one response.
☐ Man
☐ Woman
☐ Another identity not listed above. Please specify ________________
☐ I prefer not to say.

G. What language(s) do you speak most often at home?
Select all that apply.
☐ English
☐ French
☐ Arabic
☐ Cantonese
☐ Mandarin
☐ Punjabi
☐ Spanish
☐ Tagalog
☐ Another language not listed above. Please specify _____________
☐ I prefer not to answer.

H. What was your household's gross annual income last year? That is, all income from you and any other earners in your household, before taxes and other deductions?
Select one response.
☐ Less than $20,000
☐ $20,000 to less than $40,000
☐ $40,000 to less than $60,000
☐ $60,000 to less than $80,000
☐ $80,000 to less than $100,000
☐ $100,000 to less than $150,000
☐ $150,000 to less than $200,000
☐ $200,000 or more
☐ Prefer not to say.

I. What is your highest education level?
Select one response.
☐ Did not finish high school
☐ High school
☐ Post-secondary education
☐ Other. Please specify_______________
☐ Prefer not to answer.

J. Do you suffer from any of the following medical conditionss?
Select all that apply.
☐ Heart disease
☐ Cancer
☐ Lung disease
☐ Alzheimer's disease
☐ Diabetes
☐ Kidney disease
☐ Thrombosis
☐ None of the above.

MUST CHOOSE CANCER TO CONTINUE, OTHERWISE TERMINATE

Survey: Blood Clots in Patients with Cancer

Q11. What is the primary location of your cancer?
Select all that apply.
☐ Breast
☐ Stomach or esophagus
☐ Pancreas
☐ Lung
☐ Colorectal
☐ Blood (e.g., multiple myeloma, leukemia, polycythemia vera)
☐ Lymph nodes (lymphoma)
☐ Prostate
☐ Bladder
☐ Gynecologic (e.g., uterus, cervix, vagina)
☐ Skin
☐ Kidney
☐ Liver
☐ Brain

 © 2025. The Author(s).

- ☐ Melanoma
- ☐ Sarcoma
- ☐ Other. Please specify______________

Q12. What type of treatment are you receiving/have you received for your cancer?
Select all that apply.
- ☐ Chemotherapy
- ☐ Radiation therapy
- ☐ Surgery
- ☐ Targeted therapy
- ☐ Immunotherapy
- ☐ Hormone therapy
- ☐ Transplant
- ☐ None of the above.
- ☐ Not sure of the type.
- ☐ Other. Please specify______________

Q13. What is the status of your cancer treatment?
Select one response.
- ☐ Treatment starting soon
- ☐ Ongoing
- ☐ Completed
- ☐ Watch and wait
- ☐ Not sure

Q14. What is the current stage of your cancer?
Select one response.
- ☐ Cancer is in one area of the body
- ☐ Cancer has spread to other areas of the body (e.g., stage 3 or 4)
- ☐ Blood cancer is not in remission
- ☐ Blood cancer in remission
- ☐ No longer any evidence of cancer
- ☐ Unknown

Q15. When you think of blood clots, which of the following do you think of?
Select all that apply.
- ☐ Heart attack
- ☐ Stroke
- ☐ Phlebitis
- ☐ Varicose veins
- ☐ Clot in deep veins in the leg
- ☐ Clot in deep veins in the arm
- ☐ Clot in the lungs
- ☐ Clot in other parts of the body
- ☐ Aneurysm
- ☐ Hematoma
- ☐ Other. Please specify ____________

Q16. Please indicate how familiar you are with the following terms, where 1 = no knowledge and 7 = very knowledgeable.
Select one response.

No knowledge						Very knowledgeable
1	2	3	4	5	6	7

Veinous blood clots
- ☐ Deep vein thrombosis
- ☐ Venous thromboembolism
- ☐ Pulmonary embolism
- ☐ Catheter-related thrombosis
- ☐ Superficial vein thrombosis
- ☐ Superficial thrombophlebitis

Arterial blood clots
- ☐ Myocardial infarction or MI or heart attack
- ☐ Stroke
- ☐ Cerebral vascular accident
- ☐ Transient ischemic attack or TIA, sometimes called mini-stroke

Medications
- ☐ Blood thinner
- ☐ Anticoagulant
- ☐ Heparin
- ☐ Warfarin
- ☐ Aspirin
- ☐ Plavix
- ☐ Antiplatelet agent

The rest of this survey will focus specifically on blood clots that can occur in the veins, also known as thrombosis. Deep vein thrombosis (DVT) is a blood clot that occurs most commonly in the deep veins of the leg. DVTs can cause pain and swelling in the leg. If left untreated, DVTs can move to the lungs, where they cause pulmonary embolism (PE). PE can cause breathing problems, chest pain, and tiredness, and sometimes, PE can be very serious.

The part of the survey will ask you about thrombosis only. It will not be asking questions about strokes, heart attacks, or other types of blood clots.

Q17. Blood clots in people living with cancer are called cancer-associated thrombosis or CAT. Please indicate your level of knowledge about cancer-associated thrombosis, where 1 = no knowledge and 7 = very knowledgeable.
Select one response.

No knowledge						Very knowledgeable
1	2	3	4	5	6	7

Q18. Did your health care team inform you that you were more likely to get thrombosis due to your cancer diagnosis or treatment?
Select one response.
- ☐ Yes
- ☐ No
- ☐ Don't recall

Q19. Did your cancer health care team provide you with education about the signs and symptoms of thrombosis?
Select one response.
- ☐ Yes
- ☐ No
- ☐ Don't recall

IF "YES," CONTINUE. IF "NO" OR "DON'T RECALL," GO TO Q21.

 © 2025. The Author(s).

Q20. If yes, did you receive instructions on what to do or who to contact if you notice the signs and symptoms of thrombosis?
Select one response.
☐ Yes
☐ No
☐ Don't recall

Q21. Have you ever been diagnosed with thrombosis?
Select all that apply.
☐ Yes, before my cancer diagnosis
☐ Yes, after my cancer diagnosis, before I started cancer treatment (e.g., surgery, chemotherapy)
☐ Yes, during my cancer treatment (e.g., surgery, chemotherapy)
☐ No
☐ Not sure

IF SELECTED "YES," CONTINUE. IF "NO" OR "NOT SURE" GO TO Q26.

Q22. What type of thrombosis have you experienced?
Select all that apply.
☐ Deep vein thrombosis in the leg
☐ Deep vein thrombosis in the arm
☐ Deep vein thrombosis in other parts of the body (e.g., liver, brain)
☐ Pulmonary embolism
☐ Superficial vein thrombosis
☐ Catheter-related thrombosis
☐ Not sure

Q23. If you have been diagnosed with a blood clot associated with your cancer, who on the health care team told you?
Select one response.
☐ Oncologist
☐ Surgeon
☐ Hematologist
☐ Thrombosis specialist
☐ Emergency physician
☐ Radiologist
☐ Other specialist physician
☐ Family doctor
☐ Nurse/Nurse Practitioner
☐ Pharmacist
☐ Other. Please specify ___________
☐ Don't remember

Q24. Would you have wanted information about thrombosis before it happened?
Select one response.
☐ Yes
☐ No
☐ Not sure

Q25. Do you feel your quality of life changed after you had thrombosis?
Select one response.
☐ Yes
☐ No
☐ Not sure

Q26. Please indicate which of the following are signs and symptoms of thrombosis.
Select all that apply.
☐ Swelling, usually of one leg
☐ Pain in the calf, inner thigh, or groin
☐ Redness and warmth of the affected leg
☐ Lower back pain
☐ Swelling of one arm
☐ Pain along the inner arm
☐ Redness and warmth of the affected arm
☐ More noticeable/enlarged blood vessels under the skin of the affected upper arm and chest area
☐ Persistent headache
☐ Nausea
☐ Shortness of breath
☐ Chest pain
☐ Not sure

Q27. Which of the following factors or conditions have you been told or do you know will **increase** the likelihood of thrombosis in patients with cancer?
Select all that apply.
☐ Reduced physical activity
☐ Previous thrombosis
☐ Recent surgery
☐ Chemotherapy or other drug cancer therapies
☐ Radiation therapy
☐ Certain cancer types
☐ Central catheter (e.g., peripherally inserted central catheter (PICC) or Port)
☐ Poor diet
☐ Travel
☐ Family history of thrombosis
☐ Hospitalization
☐ None of the above
☐ Other. Please specify _________________
☐ I have not been told about the risk factors for thrombosis.

Q28. Has a health care professional told you there is treatment to prevent thrombosis in patients with cancer?
Select one response.
☐ Yes
☐ No
☐ Don't remember

Q29. Please rate the importance of receiving education on blood clots from your health care team, where 1 = not important and 7 = extremely important.
Select one response.

Not important						Extremely important
1	2	3	4	5	6	7

Q30. When you think of blood thinners, which of the following do you think of?
Select all that apply.
☐ Aspirin (ASA)
☐ Clopidogrel (Plavix®)

 © 2025. The Author(s).

- ☐ Warfarin (Coumadin®)
- ☐ Direct oral anticoagulants (apixaban [Eliquis®], rivaroxaban [Xarelto®], edoxaban [Lixiana®], dabigatran [Pradaxa®])
- ☐ Low-molecular-weight heparin (e.g., dalteparin [Fragmin®], tinzaparin [Innohep®], and enoxaparin [e.g., Lovenox®])
- ☐ None of the above.
- ☐ Don't know
- ☐ Other. Please specify _______________

Q31. Which anticoagulant (also known as a blood thinner) are you taking now, or have you taken in the past?
Select all that apply.
- ☐ Have never taken an anticoagulant
- ☐ Apixaban (Eliquis®)
- ☐ Dabigatran (Pradaxa®)
- ☐ Edoxaban (Lixiana®)
- ☐ Rivaroxaban (Xarelto®)
- ☐ Warfarin (Coumadin®)
- ☐ Dalteparin (Fragmin®)
- ☐ Enoxaparin (Lovenox®)
- ☐ Tinzaparin (Innohep®)
- ☐ Enoxaparin (Inclunox®)
- ☐ Enoxaparin (Noromby®)
- ☐ Enoxaparin (Redesca®)
- ☐ None of the above
- ☐ Not sure of the name

IF SELECTED "HAVE NEVER TAKEN AN ANTICOAGULANT" OR "NONE OF THE ABOVE" GO TO Q38., OTHERWISE CONTINUE

Q32. Please indicate why you are taking or have taken an anticoagulant.
Select all that apply.
- ☐ To prevent a stroke from atrial fibrillation (an abnormal heart rhythm)
- ☐ To prevent a stroke from a mechanical heart valve
- ☐ To treat or prevent a blood clot in the veins (deep vein thrombosis and/or pulmonary embolism)
- ☐ Another reason, please describe ________________
- ☐ I am not sure

Q33. Who manages your anticoagulant now? Who prescribes your refills?
Select one response.
- ☐ Family doctor
- ☐ Specialist doctor in the thrombosis clinic
- ☐ Nurse practitioner in the thrombosis clinic
- ☐ Specialist doctor not in a thrombosis clinic
- ☐ Nurse practitioner not in a thrombosis clinic
- ☐ Another health care provider
- ☐ Don't know

Q34. When the anticoagulant was first prescribed, did a health care provider (doctor, nurse, or pharmacist) discuss any of the following topics?
Select any that you remember.
- ☐ Your choice about which anticoagulant you prefer to take after describing the options.
- ☐ Why is the anticoagulant necessary?
- ☐ How many times a day to take the anticoagulant?
- ☐ The potential side effects of the anticoagulant.
- ☐ The potential benefits of the anticoagulant .
- ☐ How the anticoagulant interacts with some foods?
- ☐ How the anticoagulant interacts with other medications?
- ☐ Cost of the medication.
- ☐ Other points discussed, please explain [open text box].
- ☐ I don't remember.
- ☐ None of the above.

Q35. How comfortable do you feel taking an anticoagulant?
Select one response.

Not comfortable at all						Very comfortable
1	2	3	4	5	6	7

Q36. When you are taking an anticoagulant, do you worry about the following?
Select all that apply.
- ☐ Having more thrombosis
- ☐ Having serious bleeding
- ☐ None of the above
- ☐ Other. Please specify _______________

Q37. While taking an anticoagulant, did you experience any complications?
Select all that apply.
- ☐ More thrombosis
- ☐ Minor bleeding that did not concern me (e.g., nosebleed)
- ☐ Bleeding that was a nuisance and bothered me (e.g., heavy menstrual periods)
- ☐ Serious bleeding (e.g., needed to see a doctor or go to the emergency department)
- ☐ None
- ☐ Other. Please specify _______________

ASK ALL

Q38. What topics would you like to learn more about?
Select all that apply.
- ☐ Information about specific anticoagulants (drug information sheets)
- ☐ Information about new anticoagulants
- ☐ Information about why your doctor has prescribed an anticoagulant (disease information sheets, for example atrial fibrillation, deep vein thrombosis, pulmonary embolism, etc.)
- ☐ Benefits of anticoagulants
- ☐ Side effects of anticoagulants
- ☐ Management of anticoagulant before and after surgery/medical procedure/chemotherapy
- ☐ Screening for thrombosis
- ☐ Pregnancy and thrombosis
- ☐ Menstruation and blood thinners

 © 2025. The Author(s).

- ☐ Cost of anticoagulants
- ☐ Information about the risk of thrombosis with cancer
- ☐ Information on the risk factors for thrombosis associated with cancer
- ☐ Other, please describe _______________

Q39. What format of materials do you prefer to learn about disease or diseases?
Select all that apply.
- ☐ Videos
- ☐ Downloadable brochures
- ☐ Paper brochures
- ☐ Website
- ☐ Apps
- ☐ Other, please describe ______________

Q40. Do you know about the Thrombosis Canada website (www.thrombosiscanada.ca)?
Select one response.
- ☐ Yes
- ☐ No
- ☐ Not sure

IF "YES" CONTINUE, OTHERWISE SKIP TO Q42.

Q41. Have you ever gone to the Thrombosis Canada website for information about thrombosis or any other topic?
Select one response.
- ☐ Yes
- ☐ No
- ☐ Not sure

ASK ALL

Q42. Do you have any final comments or suggestions?
Open box

Thank you very much for completing this survey.
These are all the questions we have for you today.

Supplementary Table S1 Patients' learning topics preferences

What topics would you like to learn more about?	Percentage (*N*)
Information about the risk of thrombosis with cancer	42.6 (133)
Information on the risk factors for thrombosis associated with cancer	38.5 (120)
Side effects of anticoagulants	38.5 (120)
Screening for thrombosis	32.7 (102)
Benefits of anticoagulants	28.2 (88)
Information about specific anticoagulants (drug information sheets)	23.4 (73)
Management of anticoagulants before and after surgery/medical procedure/chemotherapy	19.6 (61)
Information about new anticoagulants	18.6 (58)
Information about why your doctor has prescribed an anticoagulant (disease information sheets, for example, atrial fibrillation, deep vein thrombosis, pulmonary embolism, etc.)	16.3 (51)
Cost of anticoagulants	10.9 (34)
Menstruation and blood thinners	7.1 (22)
Pregnancy and thrombosis	6.1 (19)
Others	5.1 (16)

 © 2025. The Author(s).

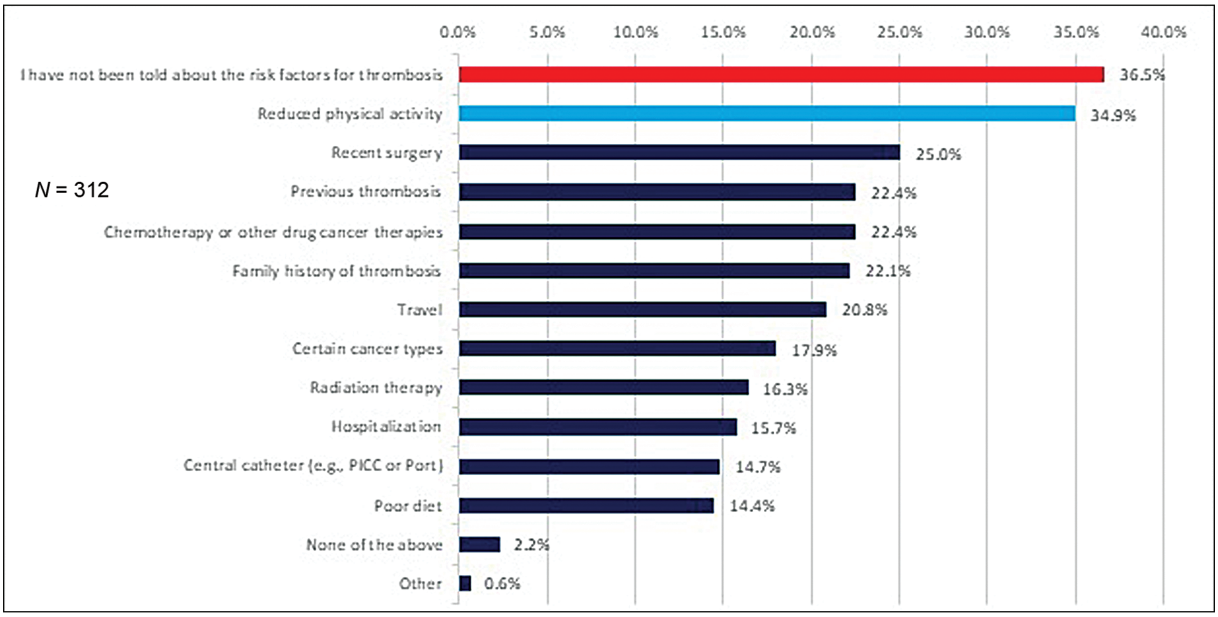

Supplementary Fig. S1 Awareness about risk factors for cancer-associated thrombosis. PICC, peripherally inserted central catheter.

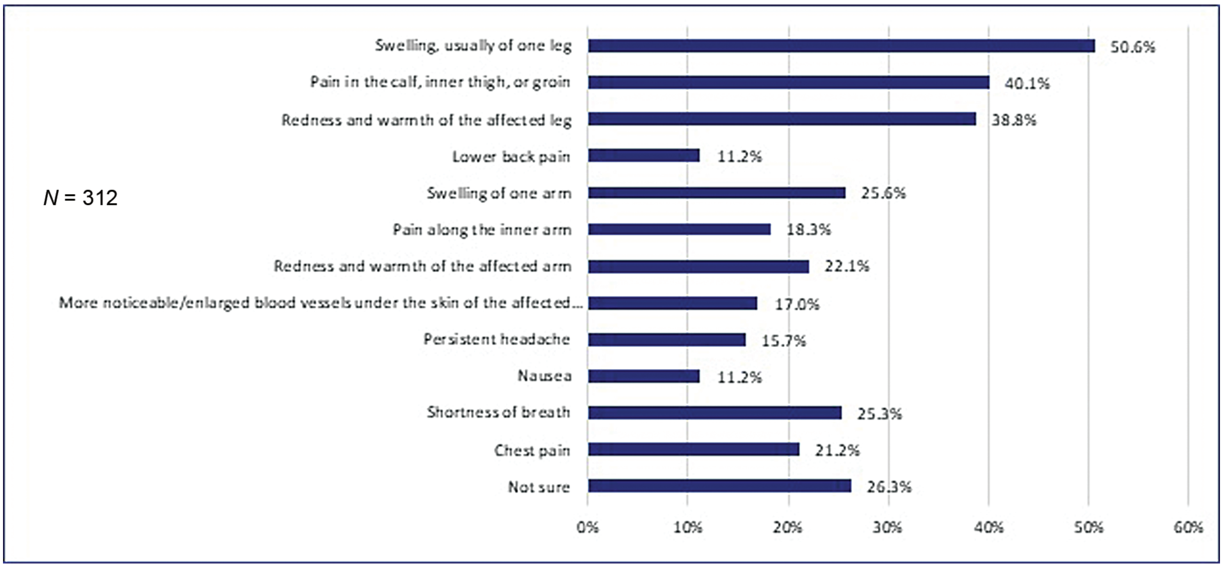

Supplementary Fig. S2 Awareness about signs and symptoms for cancer-associated thrombosis.

 © 2025. The Author(s).

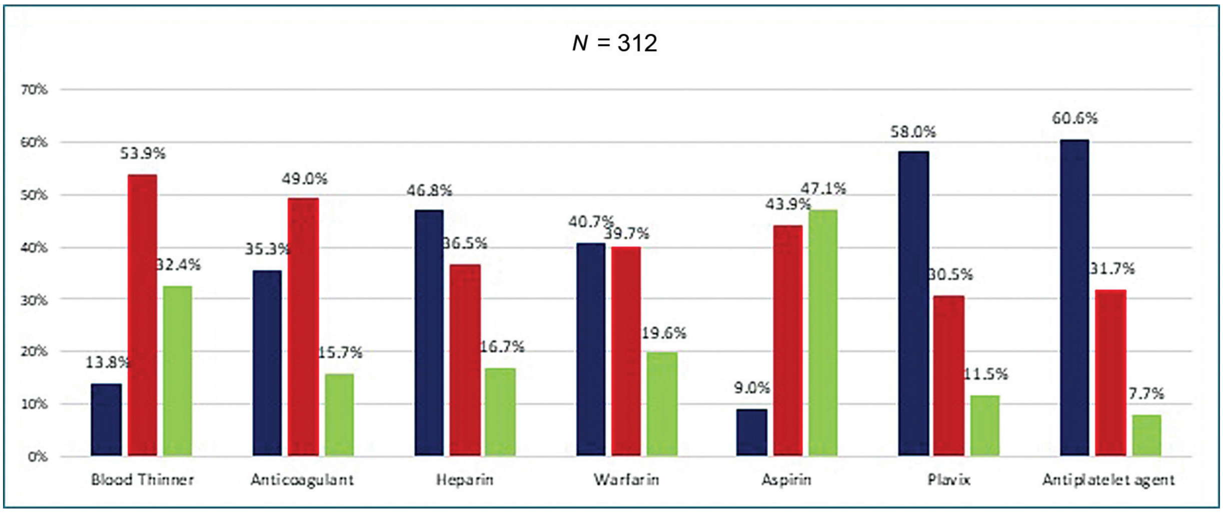

Supplementary Fig. S3 Familiarity with terms related to anticoagulant or antiplatelet agents. Respondents had to choose a number between 1 and 7 according to their familiarity with the terms proposed. Scale: 1, no knowledge to 7, very knowledgeable. Summary chart legend is green (very knowledgeable) = top two (6 and 7); red (somewhat) = middle three (3–5); blue (very little/no knowledge) = bottom two (1 and 2).

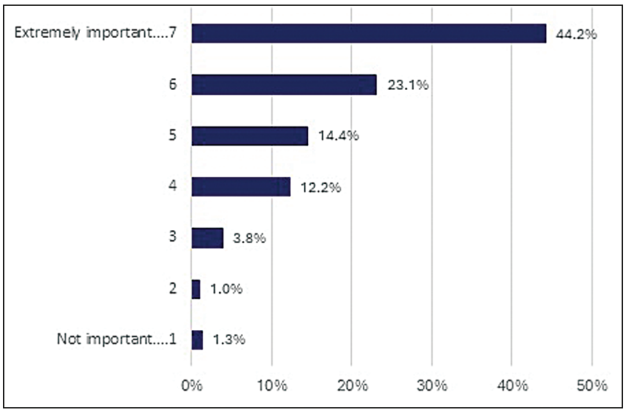

Supplementary Fig. S4 Importance of receiving education on cancer-associated thrombosis from your healthcare team. Respondents had to choose a number between 1 and 7 according to the importance they give to thrombosis education. Scale: 1, no importance to 7, extremely important. Very important = top two (6 and 7); somewhat important = middle three (3–5); very little/no importance = bottom two (1 and 2).

 © 2025. The Author(s).
